# Anti-CGRP antibody galcanezumab modifies the function of the trigeminovascular nocisensor complex in the rat

**DOI:** 10.1186/s10194-024-01717-2

**Published:** 2024-01-19

**Authors:** Nadine Friedrich, Krisztina Németh, Martin Tanner, Judit Rosta, Ildikó Dobos, Orsolya Oszlács, Gábor Jancsó, Karl Messlinger, Mária Dux

**Affiliations:** 1 Department of Physiology, University of Szeged, Dóm Tér 10, 6720 Szeged, Hungary; 2 Chemical Biology Research Group, Institute of Organic Chemistry, Research Centre for Natural Sciences, Magyar Tudósok Krt. 2, 1117 Budapest, Hungary; 3 Institute of Physiology and Pathophysiology, Friedrich-Alexander-University, 91054 Erlangen-Nuremberg, Germany

**Keywords:** Galcanezumab, Monoclonal antibody, Calcitonin gene-related peptide, Migraine

## Abstract

**Background:**

Monoclonal antibodies directed against the neuropeptide calcitonin gene-related peptide (CGRP) are effective in the prevention of chronic and frequent episodic migraine. Since the antibodies do not cross the blood brain barrier, their antinociceptive effect is attributed to effects in meningeal tissues. We aimed to probe if such an antibody can be visualized within the dura mater and the trigeminal ganglia following its administration to rats and to examine if the activity of the trigeminovascular nocisensor complex is influenced by this treatment.

**Methods:**

Effects of the anti-CGRP antibody galcanezumab on the trigeminovascular nocisensor complex was examined by measuring release of sensory neuropeptides and histamine from the rat dura mater. Deposits of galcanezumab were visualized by fluorescence microscopy in the trigeminal ganglion and the dura mater.

**Results:**

Fluorophore-labelled galcanezumab was detected in the dura mater and the trigeminal ganglion up to 30 days after treatment affirming the long-lasting modulatory effect of this antibody. In female rats, seven days after systemic treatment with galcanezumab the capsaicin-induced release of CGRP was decreased, while that of substance P (SP) was increased in the dura mater. In control rats, release of the inhibitory neuropeptide somatostatin (SOM) was higher in females than in males. Stimulation with high concentration of KCl did not significantly change the release of SOM in control animals, while in rats treated with galcanezumab SOM release was slightly reduced. Galcanezumab treatment also reduced the amount of histamine released from dural mast cells upon stimulation with CGRP, while the effect of compound 48/80 on histamine release was not changed.

**Conclusions:**

Galcanezumab treatment is followed by multiple changes in the release of neuropeptides and histamine in the trigeminal nocisensor complex, which may contribute to the migraine preventing effect of anti-CGRP antibodies. These changes affecting the communication between the components of the trigeminal nocisensor complex may reduce pain susceptibility in migraine patients treated with CGRP targeting monoclonal antibodies.

## Background

The trigeminovascular nocisensor complex of the dura mater consists of the meningeal vascular bed, the thinly myelinated Aδ and unmyelinated C fibres of primary sensory neurons, associated with dural blood vessels and meningeal immune cells; macrophages and mast cells [1–4]. The elements of this complex are anatomically and functionally interconnected and the complex is regarded as an important entity in headache generation.

The activation of meningeal nociceptors by blood- and tissue-born inflammatory agents can be involved in both peripheral and central sensitization of the nociceptive pathway [5, 6]. A significant population of meningeal afferents express nociceptive cation channels, the transient receptor potential vanilloid 1 (TRPV1) or the transient receptor potential ankyrin 1 (TRPA1) channel, which can be activated or sensitized by exogenous chemical agents or endogenous inflammatory mediators [7, 8]. A major proportion of trigeminal nociceptors are peptidergic containing calcitonin gene-related peptide (CGRP), substance P (SP), or somatostatin (SOM) [2, 9]. Changes in these peptide levels seem to play a significant role in headache generation. In the past thirty years, clinical observations firmly established the role of CGRP in migraine pathophysiology [10, 11]. Release of CGRP from the peripheral and central terminals of activated trigeminal nociceptors dilates meningeal blood vessels and may sensitize the nociceptive pathway [12, 13]. CGRP may initiate and maintain peripheral and central sensitization by modulating neuronal, glial and immune cell functions in the trigeminal nociceptive pathway. The neuropeptide SOM with an antinociceptive effect is stored in and released from primary afferents. Immunohistochemical staining showed the presence of SOM in some trigeminal neurons innervating the meningeal tissues [14]. SOM is expressed by a subpopulation of chemosensitive neurons in the trigeminal ganglion [15]. Clinical results suggested that the concentration of SOM was low interictally in migraineurs’ cerebrospinal fluid, and further reduction was measured during headache attacks [16, 17] suggesting a pain suppressing role of this peptide.

Mast cells releasing a broad spectrum of cytokines, chemokines and proteases upon stimulation play a central role in the interactions between the components of the trigeminovascular nocisensor complex [18, 19]. Neuropeptides, immunoglobulins, complement factors and other inflammatory products can trigger mast cell degranulation or induce selective release of mast cell mediators [1, 20]. These, in turn, may activate or sensitize nociceptive nerve terminals amplifying the central transmission of the nociceptive signal. In the dura mater, mast cells are the prominent source of histamine [21]. In rats, CGRP is a reliable activator of mast cells, since they express the CGRP receptor. Colocalization of mast cell tryptase and component of CGRP receptor was shown by immunohistochemistry in the rat dura mater [3].

Humanized monoclonal anti-CGRP and anti-CGRP receptor antibodies are effective in preventing headache attacks in patients suffering from chronic migraine [22, 23]. Since the penetration of the antibodies through the blood–brain barrier is limited, their migraine preventing effect is regarded to target mainly the peripheral axon and possibly the perikarya of trigeminal neurons [24]. Besides neutralizing CGRP released into the peripheral tissue, anti-CGRP antibodies may also reduce the amount of peptides released upon stimulation. Our recent experimental results showed that the anti-CGRP antibody fremanezumab is able to decrease CGRP release from meningeal afferents and to reduce the blood flow increasing effect of TRPV1 and TRPA1 receptor activation [25].

The aim of the present study was to define functional changes in the trigeminovascular nocisensor complex of rats that may affect the nociceptor function after treatment with the anti-CGRP antibody galcanezumab. To visualize the distribution of the anti-CGRP antibody in the dura mater and the trigeminal ganglion, we used fluorophore-labelled galcanezumab. An ex vivo dura mater preparation was used to examine changes in the stimulated histamine release from mast cells after anti-CGRP antibody treatment. Changes in the stimulated release of CGRP, SP and SOM from meningeal afferents was also measured after galcanezumab treatment. Provided that CGRP and SP are mainly colocalized, stored in the same vesicles and co-released upon activation [26], we also asked if treatment with a CGRP-binding antibody changes the release of SP similar to that of CGRP.

## Materials and methods

### Animals

Adult male (weighing 270–320 g) and female (230–260 g) Wistar rats were used. Rats were fed on standard diet and water ad libitum and housed under controlled conditions (12-h light/dark cycle, 22 ± 2 °C, 50—70% relative humidity). The experiments were approved by the Ethical Committee for Animal Care of the University of Szeged (approval ID: XIV./1800/2021 and XIV./2368/2023) and carried out in accordance with the Directive 2010/63/EU of the European Parliament. All efforts were made to minimize the number of animals used and their suffering.

### Administration of antibodies

Rats were anaesthetized in a plastic box with isoflurane at an increasing concentration up to 4% (Aerrane, Baxter Hungary Kft, Hungary). One group of animals received 30 mg/kg of the anti-CGRP antibody galcanezumab (in 10 mg/ml solution) via subcutaneous injection into the shaved area at the neck and shoulder region. The concentration of galcanezumab was chosen according to previously published data indicating morphological and functional changes in the rat trigeminal system following the administration of an anti-CGRP antibody at the same dose [25, 27, 28]. Galcanezumab was taken from the commercial injector containing 120 mg galcanezumab (Emgality, Eli Lilly Netherlands B.V., Utrecht, Nederland) and diluted with saline (0.9% NaCl). Control rats received equivalent amounts of the vehicle (0.9% NaCl).

To visualize the presence and the distribution of galcanezumab in the dura mater and the trigeminal ganglion, a fluorophore-labelled antibody was used (see below). For comparison, fluorophore-labelled bevacizumab (30 mg/kg in 10 mg/ml solution Avastin, Roche, Switzerland), a humanized monoclonal anti-tumor antibody targeting the vascular endothelial growth factor, was administered subcutaneously in some experiments.

### Labelling of antibodies with fluorophore

As a first step in the fluorescent modification of galcanezumab and bevacizumab, the original buffer (not defined by the manufacturer) was exchanged to 110 mM NaHCO_3_/Na_2_CO_3_, pH 9.0 using desalting Zeba™ spin column (7 K MWCO; # 89,890 Thermo Scientific USA) according to the manufacturer’s protocol. Five equivalents of Cy3-active ester (succinimidyl ester, NHS-Cy3) was added to the IgG antibodies (galcanezumab: 120 mg/ml; 800 μM; bevacizumab: 25 mg/ml; 167 μM) in two portions, each time reacting at room temperature in the dark for 30 min. The excess reagents were removed, and buffer was exchanged with sterile and isotonic Salsol solution by using the above mentioned Zeba™ spin column. Reaction yields, IgG concentration, and purity were checked by two types of capillary electrophoresis methods and intact protein mass spectrometry analysis [29].

### Immunohistochemical staining of the dura mater and the trigeminal ganglion

Rats injected with the fluorophore-labelled galcanezumab (Cy3-galcanezumab) or bevacizumab (Cy3-bevacizumab) 3 or 30 days prior to the experiment were deeply anaesthetized with thiopental sodium (200 mg/kg, intraperitoneally, Braun, Spain) and perfused transcardially with physiological saline followed by 4% paraformaldehyde in phosphate buffer (pH 7.4). The animals were decapitated, skin and muscles were removed and the skull was divided into halves along the sagittal suture. After removing the brain, the parietal dura mater and the trigeminal ganglia were dissected and postfixed for 2 h in the same fixative. Then trigeminal ganglia were placed in 0.1 M phosphate buffered saline (pH 7.4) containing 30% sucrose at 4 °C for 24 h and cut into 16 μm thick longitudinal sections using a cryostat (Leica CM 1950, Switzerland).

Whole mount preparation of the dura mater and sections of trigeminal ganglia of Cy3-galcanezumab-treated animals were processed for staining with the indirect immunofluorescence technique using a monoclonal mouse anti-CGRP antibody (1:500, Sigma-Aldrich, Germany), a monoclonal mouse anti-smooth muscle actin antibody (1:1000, Sigma Aldrich, Taufkirchen, Germany), a polyclonal rabbit anti-von Willebrand factor antibody (1:50, abcam, Cambridge, UK) or a polyclonal rabbit anti-histamine antibody (1:100, GeneTex, USA). IgG labelled with DyLight 488 or Alexa 488 was used as secondary antibody (both 1:500, Jackson Immunoresearch Laboratories, USA). Some sections of trigeminal ganglia were mounted with Roti-Mount FluorCare DAPI (Sigma Aldrich, Taufkirchen, Germany). Preparations of the dura mater and trigeminal ganglia were examined with a confocal fluorescence microscope (ZEISS LSM 700, Germany).

### Ex vivo measurement of CGRP, SP and SOM release from meningeal afferents

Measurement of CGRP, SP and SOM release from the rat dura mater was performed by the method originally developed by Ebersberger et al [30]. Control male and female rats treated with galcanezumab or vehicle 7 days prior to the experiment were deeply anaesthetized with thiopental sodium (200 mg/kg, intraperitoneally) and decapitated. After removal of the skin and muscles, the skull was divided into halves along the midline and the cerebral hemispheres were removed. The skull preparations were washed with carbogen-gassed synthetic interstitial fluid (SIF, containing in mM: NaCl 135, KCl 5, MgCl_2_ 1, CaCl_2_ 5, glucose 10 and Hepes 10, pH 7.4) at room temperature for 30 min and then mounted in a humid chamber at 37 °C. The cranial fossa was filled with 300 μl of carbogen-gassed SIF solution. Samples of the superfusate were collected at periods of 10 min. Control samples were taken to determine basal peptide release in the presence of SIF, then the dura was stimulated for 10 min with the TRPV1 receptor agonist capsaicin at 100 nM in case of CGRP and SP or with 60 mM KCl in case of SOM release. 200 μl of samples diluted with 50 μl of enzyme-linked immunoassay (EIA) buffer were placed into Eppendorf cups and immediately frozen at -70 °C for subsequent analysis. The EIA method was used for the measurement of CGRP (Bertin Pharma, France), SP and SOM content (MyBioSource, USA) of the defrosted samples. For CGRP and SP measurements the same samples divided into halves (125 μl) were used. Peptide concentrations of the superfusates were expressed in pg/ml. Changes in peptide release were expressed as percentage changes relative to the basal release.

### Ex vivo measurement of histamine release from meningeal mast cells

Skull halves of control rats and animals treated with galcanezumab 7 days prior to the experiment were prepared as described above for the measurement of peptide release. Control samples were taken in the presence of SIF for 10 min to determine basal histamine release, then the dura mater was stimulated for 10 min by application of 300 μl of CGRP (Sigma-Aldrich, Germany) at 10 μM or 2.5 μg/ml compound 48/80 (Sigma-Aldrich, Germany). The concentrations of CGRP and compound 48/80 used in the experiments were found effective in releasing histamine in previous experiments of our laboratory [31]. 100 μl of samples diluted with 25 μl of EIA buffer were placed into Eppendorf cups and immediately frozen at -70 °C for subsequent analysis. The EIA method was used for measurement of the histamine concentration (Bertin Pharma, France) in pg/ml. Changes in release were expressed as percentage changes relative to the basal values.

### Statistics

Statistical analysis was performed using Statistica 13 software (StatSoft, USA). Following verification of the normal distribution of data, the Student’s t-test and analysis of variance (factorial ANOVA or one-way ANOVA) extended by the unequal *N* honest significant difference (HSD) test were used as specified in the results. All values were expressed as mean ± standard error of the mean (SEM). A probability level of *p* < 0.05 was regarded as statistically significant.

## Results

### Fluorophore-labelled antibodies are located in the wall of arterioles and the dural connective tissue

**Fig. 1:**
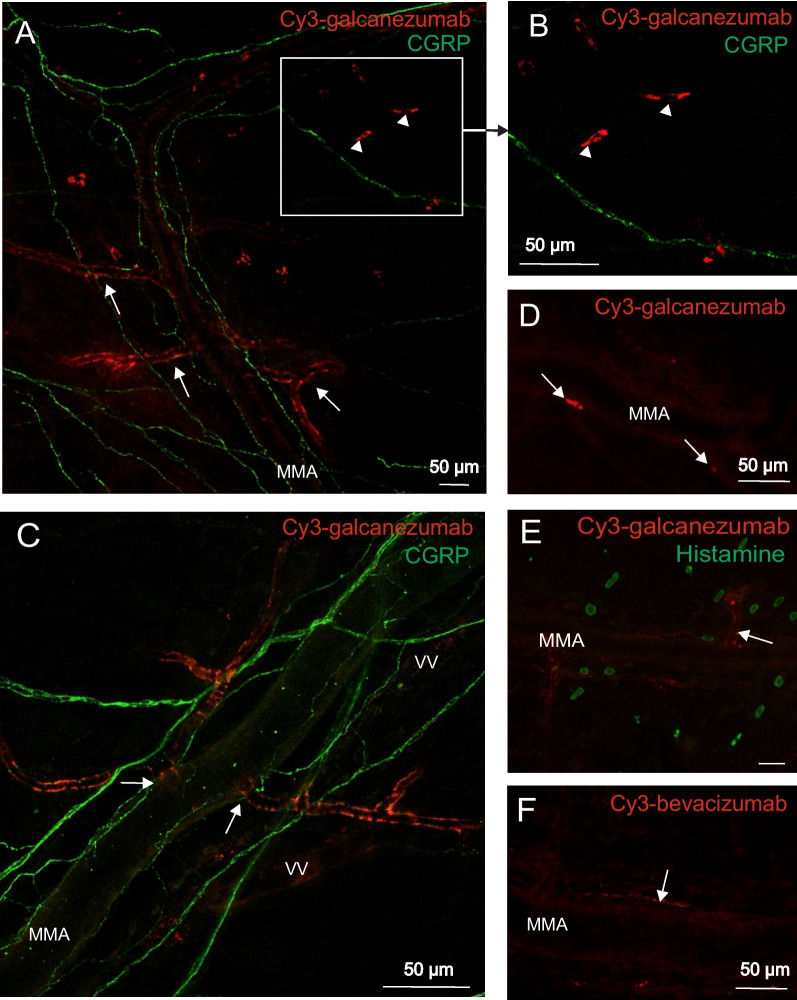
Immunohistochemical localization of Cy3-galcanezumab (**A-E**) and Cy3-bevacizumab (**F**) in whole mount preparations of the rat dura mater 3 days (**A-C**, **E–F**) or 30 days (**D**) after injection of the antibodies. Arrows indicate blood vessels, arrowheads (**A-B**) cells with deposition of fluorophore-labelled antibody galcanezumab. MMA: middle meningeal artery, VV: venous vessel

In whole mount dura mater preparations from animals treated with Cy3-galcanezumab 3 days prior to fixation, the fluorescence signal was detected mainly in the wall of small branches of the middle meningeal artery with a diameter of 20–30 μm, which can be assigned mainly to arterioles (Fig. 1A,C). Larger branches of the arteries and capillaries were free from Cy3-fluorescence but traces of the antibody could be seen in some larger venous blood vessels (Fig. 1C). Arterioles showing the fluorescence signal were in close vicinity to trigeminal CGRP-positive afferent nerves (Fig. 1A,C). In addition, some cells in the connective tissue of the dura mater distant from visible blood vessels showed Cy3-fluorescence (Fig. 1A,B). Mast cells were identified by their histamine immunofluorescence, which was not colocalized with Cy3-galcanezumab labelling. Mast cells were mainly located in close proximity to Cy3-positive blood vessels (Fig. 1E).

**Fig. 2:**
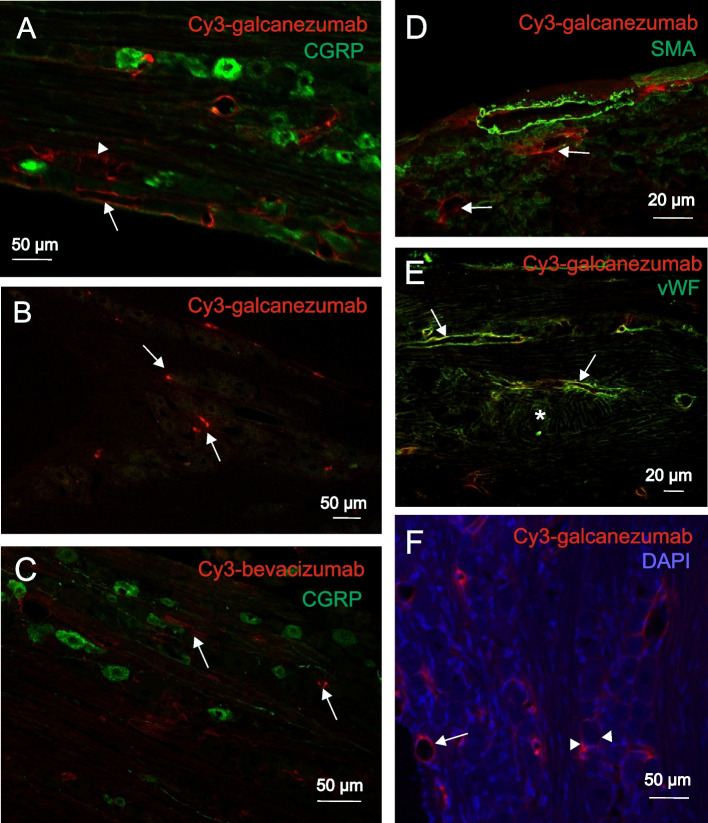
Immunohistochemical localization of Cy3-galcanezumab (**A-B**, **D-F**) and Cy3-bevacizumab (**C**) in rat trigeminal ganglia 3 days (**A**, **C-F**) or 30 days (**B**) after injection of the antibodies. Arrows indicate blood vessels, arrowheads (**A**,**F**) ganglion cells with deposition of fluorophore-labelled galcanezumab. Around * in panel E, capillaries labelled with the anti-von Willebrand factor (vWF) antibody can be seen. SMA: smooth muscle actin

Similar to the dura mater, in sections of the trigeminal ganglia Cy3-fluorescence, indicating galcanezumab, was localized in the wall of blood vessels close to CGRP-immunopositive trigeminal neurons (Fig. 2A). The fluorescence signal was also detected around the soma of some trigeminal ganglion cells (Fig. 2A,F). The fluorescence signal in the blood vessels was not colocalized with the fluorescence marker for smooth muscle actin (Fig. 2D) but in almost all labelled blood vessels with the endothelial marker for von Willebrand factor (Fig. 2E). Capillaries positive for von Willebrand factor (marked by * in Fig. 2E) did not show Cy3-fluorescence for galcanezumab.

Traces of Cy3-galcanezumab were still present in the dura mater and the trigeminal ganglion 30 days after the injection of the antibody (Fig. 1D, 2B). In our experiments, Cy3-bevacizumab was used as a control antibody without CGRP targeting property. Although the localization of Cy3-bevacizumab in the blood vessels of the dura mater and the trigeminal ganglia was similar to that of Cy3-galcanezumab, deposition of the antibody around the soma of neurons in the ganglion was absent and the labelling was overall visibly less intense (Fig. 1F, 2C).

### Galcanezumab treatment alters CGRP and SP release from the dura mater

#### Basal CGRP and SP release

Using hemisected skull preparations, we measured the concentrations of CGRP and SP released during application of the TRPV1 receptor agonist capsaicin (100 nM) in the same samples. In control animals, basal CGRP release was lower in males (19.32 ± 0.46 pg/ml, *n* = 14) than in females (23.48 ± 1.23, *n* = 10, t-test, *p* < 0.005), while SP concentrations measured in the same samples tended to be higher in males (6.17 ± 0.77 pg/ml) than in females (5.74 ± 1.24 pg/ml, t-test, *p* = 0.76).

#### Stimulated CGRP release

**Fig. 3:**
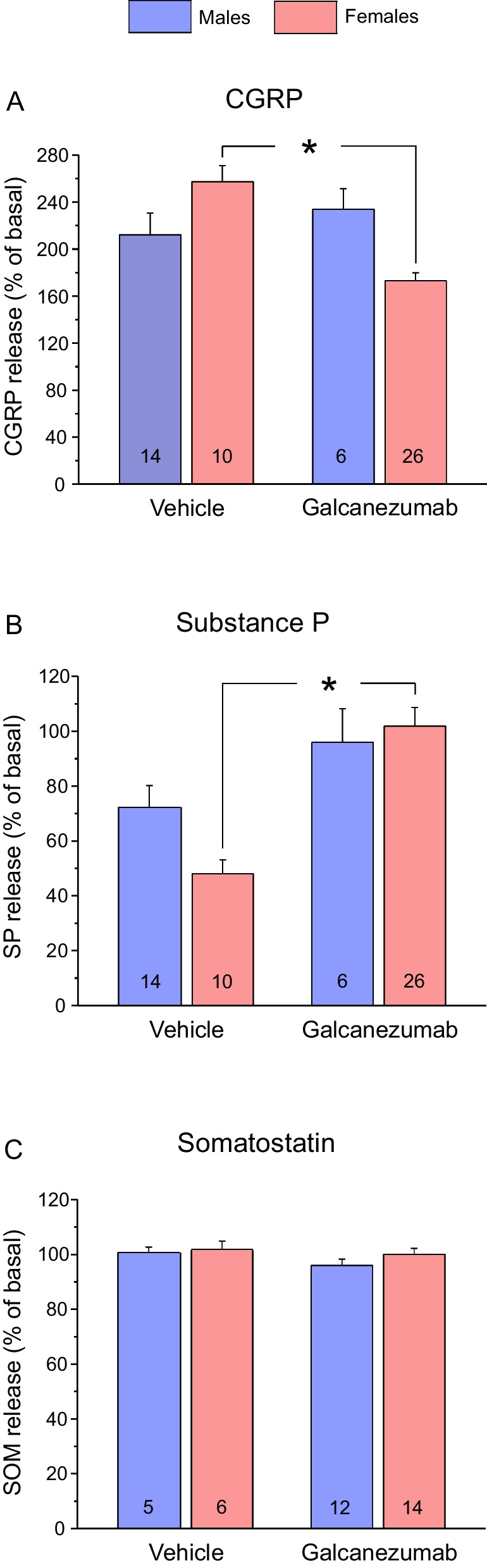
Relative changes in CGRP, SP and SOM release in vehicle-treated animals and in animals 7 days after treatment with galcanezumab. CGRP and SP content was measured in the same sample after stimulating the dura mater with capsaicin at 100 nM, while release of SOM was induced with KCl at 60 mM. The number of experiments is indicated in the bars. *: *p* < 0.05

In control animals treated with vehicle (*n* = 24), capsaicin stimulation increased the CGRP release to 231.02 ± 12.78% of the basal release. In galcanezumab-treated animals (*n* = 32), the stimulated increase in CGRP release was 184.50 ± 7.60%, which was significantly different from the vehicle group (factorial ANOVA, *F*_*1,52*_ = 4.60, *p* < 0.05). There was no significant difference between the sexes (*F*_*1,52*_ = 0.285, *p* = 0.595) but post-hoc testing using the unequal *N* HSD test showed that the difference between the vehicle and the galcanezumab group was solely due to the female animals (Table 1, Fig. 3A).

**Table 1:** Sex-specific releative changes in capsaicin-stimulated CGRP and substance P release

	Stimulated CGRP release				Stimulated substance P release			
Sex	Males		Females		Males		Females	
Treatment group	Vehicle (*n* = 14)	Galcan. (*n* = 6)	Vehicle (*n* = 10)	Galcan (*n* = 26)	Vehicle (*n* = 14)	Galcan. (*n* = 10)	Vehicle (*n* = 6)	Galcan. (*n* = 26)
Mean (%)	212.19	233.89	257.39	173.10	72.30	95.99	47.96	101.86
SEM (%)	18.32	17.45	13.81	6.81	7.84	5.18	12.22	10.80
*P*_*HSD*_	0.858		< 0.005		0.770		< 0.05	

*P*_*HSD*_ values are from unequal *N* honest significance post-hoc test following factorial ANOVA with factors treatment and sex*.* *Galcan.* Galcanezumab

#### Stimulated SP release

The capsaicin-stimulated SP release was below the basal release in vehicle-treated animals (62.16 ± 5.56%) but not different from the basal release in galcanezumab-treated animals (100.76 ± 9.0%), which was indicated as significant between the groups (factorial ANOVA, *F*_*1,52*_ = 8.847, *p* < 0.005). There was no significant difference between the sexes (*F*_*1,52*_ = 0.501, *p* = 0.482). According to the unequal *N* HSD post-hoc test, the difference between the control and the galcanezumab group was solely due to the female animals (Table 1, Fig. 3B).

### Galcanezumab treatment does not change somatostatin release from the dura mater

In other hemisected skull preparations we measured the SOM release from the dura mater stimulated with 60 mM KCl. In vehicle-treated control animals both basal and KCl stimulated SOM release tended to be higher in females. In males the basal release was 18.68 ± 0.03 pg/ml (*n* = 5), in females it was 19.16 ± 0.16 pg/ml (*n* = 6, t-test, *p* < 0.05). Stimulation in control animals caused no significant changes in SOM release (Table 2, Fig. 3C). Factorial ANOVA showed no significant difference between the treatments (*F*_*1,33*_ = 1.0, *p* = 0.332) and the sexes (*F*_*1,33*_ = 0.0, *p* = 0.889).

**Table 2:** Sex-specific relative changes in KCl-stimulated somatostatin release

	Stimulated somatostatin release			
Sex	Males		Females	
Treatment group	Vehicle (*n* = 5)	Galcan. (*n* = 6)	Vehicle (*n* = 10)	Galcan. (*n* = 14)
Mean (%)	100.41	97.63	100.80	99.71
SEM (%)	0.29	0.68	0.61	0.17

*Galcan.* Galcanezumab

### Galcanezumab treatment differentially alters the histamine releasing effect of mast cell degranulating agents

**Fig. 4:**
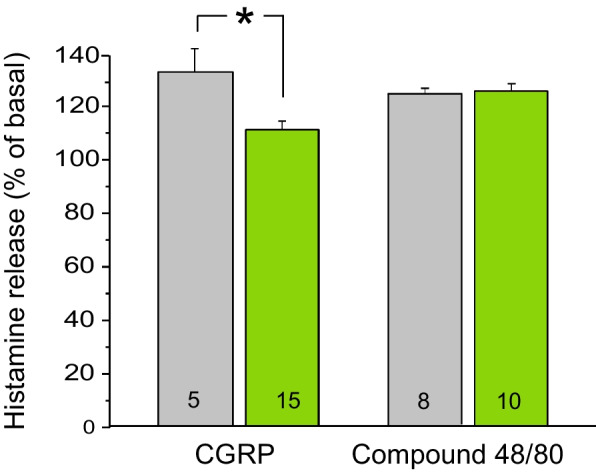
Relative changes in histamine release induced by CGRP at 10 μM and 2.5 μg/ml compound 48/80 in control animals (grey) and rats treated with galcanezumab 7 days prior to the experiment (green). The number of experiments is indicated in the bars. *: *p* < 0.05

In other hemisected skull preparations of male rats, we measured histamine release from the dura mater. The basal histamine release was 31.39 ± 1.88 pg/ml in vehicle-treated (*n* = 13) and 36.71 ± 1.05 pg/ml in galcanezumab-treated animals (*n* = 25), which was significantly different (t-test, *p* < 0.05). The histamine-releasing effects of mast cell degranulating agents were differentially influenced by galcanezumab treatment. In vehicle-treated control animals, CGRP (10 μM) increased the histamine release to 134.9 ± 7.5% (*n* = 5) of the basal release, while 7 days after treatment with galcanezumab the increase was 110.5 ± 4.7% (*n* = 15), which was significantly different from the control (one-way ANOVA for treatment, extended by the unequal HSD test, *F*_*1,18*_ = 7.03, *p* < 0.05). The histamine-releasing effect of the widely used mast cell degranulating agent compound 48/80 at 2.5 μg/ml was not different between vehicle (124.6 ± 6.2% of basal, *n* = 8) and galcanezumab-treated animals (126.4 ± 6.7% of basal, *n* = 10, one-way ANOVA, *F*_*1,16*_ = 0.04, *p* = 0.85; Fig. 4).

## Discussion

The present study was initiated in an attempt to examine changes in activity parameters of the trigeminal nocisensor complex influenced by the systemic administration of the humanized monoclonal anti-CGRP antibody galcanezumab. The parameters measured, release of CGRP as well as histamine from the dura mater, are regarded as relevant for headache generation in migraineurs. Rats were subcutaneously treated with the anti-CGRP antibody in a manner similar to the clinical use for the prevention of chronic migraine. Galcanezumab treatment was well tolerated by the rats and did not cause any visible change in their behaviour.

### Labelling with galcanezumab in the dura mater and the trigeminal ganglion

Cy3 binding to lysine amino acids of the immunoglobulin could be detected in the trigeminovascular system for up to 30 days after the administration. In the labelled arterioles, Cy3-galcanezumab was coexpressed to a great extent with the endothelial marker von Willebrand factor but not with smooth muscle actin. We assume that the strong affinity of some components of the luminal glycocalyx of endothelial cells of dural blood vessels to the circulating antibody may form a local depot that releases antibody for at least 30 days after the injection [32].

Although the exact mechanism is unclear, the high affinity of the antibody to arterioles can be explained by the diverse gene expression of adhesion molecules along the vascular tree [33]. The luminal surface of vascular endothelial cells is covered by membrane-bound, negatively charged proteoglycans, glycolipids and glycosaminoglycans, contributing to mechanotransduction, cell signaling and adhesion of blood components. We assume that the specific properties of endothelial cells are responsible for the selective accumulation of galcanezumab at specific blood vessels [33]. Since we do not know the exact mechanism of the interaction between the fluorophore-labelled anti-CGRP antibody and the vascular endothelium, we also tested the distribution of fluorophore-labelled bevacizumab, a clinically approved anti-angiogenic antibody directed against vascular endothelial growth factor. Although both antibodies could be identified in blood vessels of the dura mater and the trigeminal ganglion, the intensity and the exact localisation of labelling were different. Our finding makes it unlikely that Cy3 label alone could be responsible for the accumulation and characteristic distribution of the antibody at the endothelial cells.

Although the dose of galcanezumab used in our experiments was higher than the initial dose normally used for preventive migraine therapy, the slow elimination and the repeated administration of the antibody in humans may increase its concentration in trigeminal tissues [34, 35].

In an earlier study, the presence of the anti-CGRP antibody fremanezumab in the meningeal tissue of rats was visualized [27]. In this study, a very similar distribution of the fluorophore-labelled antibody was seen in the wall of meningeal blood vessels. Similar to our observations, they detected the fluorescence signal around the soma of trigeminal neurons already four hours after the injection of the antibody. Although the anti-CGRP antibody used (fremanezumab vs galcanezumab) and also the way of administration (intravenous vs subcutaneous) were different, the very similar distribution of the antibodies in the trigeminovascular system suggests a common fate of the two anti-CGRP antibodies in the trigeminovascular system.

Although the presence of the anti-CGRP antibody was robust both in the dura mater and the trigeminal ganglion already 3 days after the Cy3-galcanezumab injection, the functional tests were performed a few days later (7 days after the injection) to allow possibly complete functional changes in the nocisensor complex.

### CGRP and SP release from rat dura mater, sex differences

Based on earlier observations indicating that CGRP and SP are largely stored in the same vesicles and co-released upon activation of the afferents [26, 36], we asked if treatment with an anti-CGRP antibody changes the SP release from the dura mater in hemisected rat skulls in the same way as the CGRP release. Our experiments showed reduced CGRP release from meningeal afferents after treatment with the anti-CGRP antibody that affirms our earlier observations regarding the reduced CGRP releasing and blood flow increasing effect of capsaicin in the dura mater of fremanezumab-treated animals [25]. There is evidence that the anti-CGRP antibody fremanezumab reduces CGRP production in trigeminal neurons of rats [28] and we speculate that this may be similar after galcanezumab treatment, since the capsaicin-induced release of CGRP was reduced by galcanezumab treatment. The clinical relevance for an increased SP production/release concomitant with a decreased CGRP production/release is unclear, while the involvement of SP in migraine pathophysiology is a matter of speculation [37].

Migraine is more frequent in the female population. Our results reveal a sex difference not only in the basal but also in the capsaicin-induced CGRP release. The higher susceptibility of the female trigeminal system to release CGRP is also reflected by the stronger inhibition on CGRP release after galcanezumab treatment.

In galcanezumab-treated animals, capsaicin-induced CGRP and SP content of the samples was inversely related. TRPV1 receptor activation-induced SP release was more intense after anti-CGRP antibody treatment in both sexes and the difference was significant in females. Our results raises the notion that less CGRP production in the neuron may set the machinery free that leads to an increased SP production or transport to the vesicles.

### Somatostatin release from meningeal afferents and sex differences

SOM released from activated peripheral terminals of capsaicin-sensitive primary afferent neurons inhibits not only the acute inflammation and nociception but exerts a “sensocrine” function with systemic antiinflammatory and analgesic effects [38]. Immunohistochemical studies have demonstrated the presence of SOM within trigeminal neurons [39–41] and a partial coexistence with SP in the human trigeminal ganglion [14]. One of our hypotheses was that the migraine-preventing effect of the anti-CGRP antibodies may not only be due to reduced CGRP expression and effectivity. Thus it appeared possible that enhanced expression and release of SOM with its systemic pain inhibiting effect is also involved. Capsaicin has been shown to induce SOM release in different tissues but only about 8% of trigeminal ganglion neurons express this neuropeptide [40, 42]. Therefore, to ascertain a measurable effect in SOM release, we used KCl instead of capsaicin as a potent depolarizing stimulus. Our results indicated sex differences for basal SOM release in control animals with a significantly higher release in females compared to males. However, basal as well as stimulated SOM concentrations were very similar in both vehicle and galcanezumab-treated animals suggesting that it is unlikely that an increased SOM release may contribute to a pain reducing effect of galcanezumab treatment.

### Histamine release from rat dura mater

In the preparations of the dura mater we could not find any colocalization between Cy3-galcanezumab and immunohistochemically detected histamine, but the close proximity of labelled blood vessels, CGRP immmunoreactive afferents and mast cells may provide a morphological basis for a mast cell modifying effect of CGRP. Although CGRP released histamine from mast cells only at high concentrations [31], this appears possible regarding the close apposition of CGRP releasing sites and mast cells. Hence, partial neutralization of released CGRP by galcanezumab may prevent this signalling.

The role of histamine released by mast cells is not clear in migraine pathophysiology. Although histamine infusion generates pulsating headache in migraine patients, treatment with antihistamines seems to be uneffective in alleviating migraine attacks [43]. After all, the contribution of activated mast cells in the vitious circle of nociceptor activation leading to sensitization of the nociceptive pathway cannot be ruled out. In our experiments histamine release was measured as an indicator of mast cell activation. We tested two different degranulating agents acting on different G-protein coupled mast cell receptors. CGRP acts on its canonical receptor consisting of receptor activity-modifying protein 1 (RAMP1), calcitonin receptor-like receptor (CLR) and the intracellular receptor component protein (RCP). Compound 48/80 acts on Mas-Related G-Protein-Coupled Receptor Member X2. The finding of differential effects of mast cell degranulating agents on histamine release suggests that galcanezumab treatment modifies the signalling not by altering the histamine content of mast cells but most likely at or beyond the level of membrane receptors.

We do not know the exact mechanism of the difference between the basal histamine release in control (vehicle-treated) and galcanezumab-treated animals. The higher concentration of spontaneously released histamine measured in galcanezumab-treated animals may be due to changes in the number/function of mast cell receptors stimulated by tissue metabolites under basal conditions. In a previous study of our laboratory we found that after treatment of the animals with another anti-CGRP antibody, fremanezumab, the fraction of trigeminal ganglion neurons immunoreactive to CGRP and the CGRP receptor components CLR and RAMP1 was significantly lowered compared to the control [28]. Our present results indicate that changes in the CGRP receptor components induced by the antibody treatment affects not only neurons but also other components of the trigeminal nocisensor complex such as mast cells.

Although a limitation of our study is the lack of information about histamine releasing effects of CGRP and compound 48/80 in mast cells of female animals, we can expect a similar, possibly even more robust change than what we have measured in male animals after galcanezumab treatment. Anti-CGRP antibody treatment-induced functional changes (e.g. release of neuropeptides) are typically more robust in female animals [25].

## Conclusions

Our experiments detected a multi-faceted change in the function of the trigeminovascular nocisensor complex after galcanezumab treatment. Anti-CGRP antibody treatment seems to change CGRP release from trigeminal neurons upon their stimulation. Expression and release of other neuropeptides may also be changed by the antibody treatment. Activating effects of CGRP on non-neural elements of the trigeminal nocisensor complex, notably the dural mast cells, seem to be mitigated. A shift in the equilibrium between pronociceptive and antinociceptive mediators released upon trigeminal activation may contribute to the beneficial effect of anti-CGRP antibody treatment in migraine.

## Data Availability

All data generated or analysed during this study are included in this published article.

## Abbreviations

CGRP: Calcitonin gene related peptide
EIA: Enzyme-linked immunoassay
SEM: Standard error of the mean
SIF: Synthetic interstitial fluid
SOM: Somatostatin
SP: Substance P
TRPA1: Transient receptor potential ankyrin 1
TRPV1: Transient receptor potential vanilloid 1
